# Recent Trends in the Antidiabetic Prominence of Natural and Synthetic Analogues of Aurones

**DOI:** 10.3390/cimb45100533

**Published:** 2023-10-19

**Authors:** Rammohan Aluru, Anindita Mukherjee, Grigory V. Zyryanov, Adinath Majee, Sougata Santra

**Affiliations:** 1 Chemical Engineering Institute, Ural Federal University, 19 Mira Str., 620002 Yekaterinburg, Russia; rammohan4ever@gmail.com (R.A.); anindita0423@gmail.com (A.M.); gvzyryanov@gmail.com (G.V.Z.); 2 I. Ya. Postovsky Institute of Organic Synthesis of RAS, Ural Division, 22/20 S. Kovalevskoy/Akademicheskaya Str., 620219 Yekaterinburg, Russia; 3 Department of Chemistry, Visva-Bharati (A Central University), Birbhum, Santiniketan 731235, India; adinath.majee@visva-bharati.ac.in

**Keywords:** aurones, antidiabetic, chalcone, α-glucosidase inhibitor, flavonoids

## Abstract

Natural products are a boundless source for the development of pharmaceutical agents against a wide range of human diseases. Accordingly, naturally occurring aurones possess various biological benefits, such as anticancer, antioxidant, antimicrobial, antidiabetic, anti-inflammatory, antiviral and neuroprotective effects. In addition, various studies have revealed that aurones are potential templates for the regulation of diabetes mellitus and its associated complications. Likewise, certain aurones and their analogues have been found to be remarkable kinase inhibitors of DARK2, PPAR-γ, PTPM1, AGE, α-amylase and α-glucosidase, which represents a promising approach for the treatment of chronic metabolic disorders such as diabetes. Therefore, in our present study, we provide a detailed account of the advances in aurones as antidiabetic agents over the past decade.

## 1. Introduction

Among the world’s fastest-growing non-communicable diseases, diabetic mellitus (DM) is the foremost chronic metabolic disorder, threatening people’s lives and resulting in economic burden. According to a study by the International Diabetic Federation (IDF), approximately 552 million people might be suffering from diabetes worldwide by the year 2030 [1,2]. Blood glucose intolerance is the main reason for the chronic metabolic disorder called “diabetic mellitus”. The disorder can be classified into three categories: type-I DM is due to insufficient insulin secretion triggered by mechanical failure of the pancreas, and type-II DM occurs due to insulin resistance. Gestational and neonatal diabetes belong to the third category of DM [3,4]. In general, type-II DM is very common, and it is induced by lifestyle habits and hereditary factors [5]. Currently, various types of drugs, such as sulfonylureas, biguanides, thiazolidinediones, α-glucosidase inhibitors, meglitinides, GLP-1 mimetics, DPP-IV inhibitors, SGLT2 inhibitors, etc., are available to treat diabetes and associated mechanisms [6]. However, medicines currently in use have various moderate to lethal adverse effects, such as dehydration, diarrhea, constipation, bloating, nausea, gastrointestinal problems, kidney disease, respiratory tract infections, coronary artery diseases (CAD), dermatological problems, and injection site infections, etc. In addition, the number of people being diagnosed with diabetes is also increasing massively. Therefore, new therapeutic approaches and amplified drugs are needed to tackle this complex-patterned metabolic disorder.

In this regard, natural products are a prominent resource for modern drug discovery, having already provided a range of therapeutic drugs [7,8]. For instance, broad-spectrum antibiotics such as *β*-lactam, tetracycline, ciprofloxacin and erythromycin are still important clinical drugs of choice for various diseases today [9,10]. In addition, people are now aware of the role of natural antioxidants in the prevention of various non-communicable diseases and of their health promoting benefits [11,12]. Therefore, fruits and food beverages that are richer in polyphenolics such as anthocyanins, catechins, phenolic acids, flavonoids, stilbenes and resveratrols have strategic key roles in health promotion and disease prevention [12,13,14]. 

**Figure 1:**
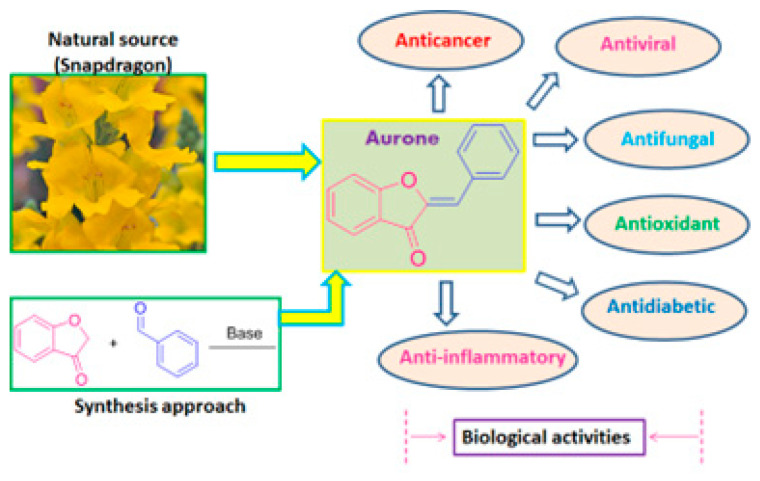
Schematic representation of the source of aurones and their biological importance.

Interestingly, aurones [2-benzylidenebenzofuran-3(2*H*)-ones] are naturally occurring five-membered flavonoids with benzofuran class heterocycles having benzylidene moiety at C-2. In the last decade, they have been recognized as a template for diverse pharmacological activities (Figure 1), such as antioxidant, antimicrobial, antimalarial, antitumor, antidiabetic and neuroprotective capabilities [15]. Moreover, a recent study also summarized the potential for using aurone scaffolds as markers in the preventive and therapeutic mechanisms of various cancers [16]. Accordingly, aurone scaffolds exhibit a wide range of anticancer properties through various modes of action, such as adenosine receptor, cyclic dependent kinase, DNA scissoring, histone deacetylase, sirtuins, topoisomerase, tubulin, tyrosinase, TNFα, PEG2 and nitric oxide inhibitory mechanisms. However, there have been no significant comprehensive studies on the antidiabetic potentialities of aurones. Therefore, in the current study, we provide a focused account of advances in the use of aurones in the amelioration of glucose metabolisms and in antidiabetic drug development. 

## 2. Occurrence and Distribution of Aurones

**Figure 2:**
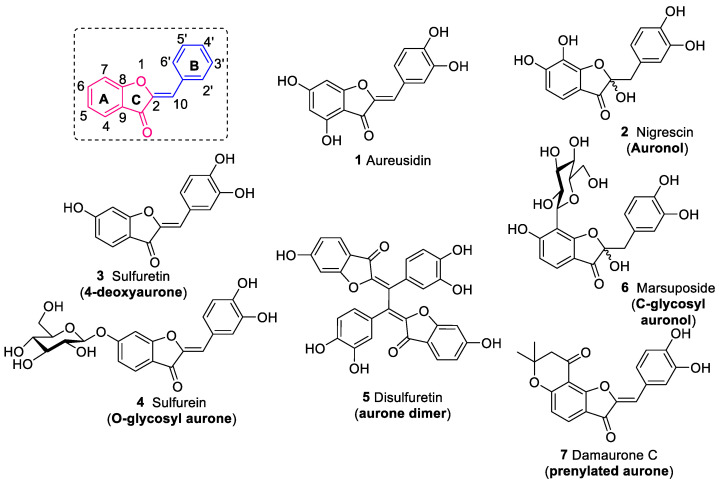
Common numbering of aurone rings and some interesting natural examples. Pink color represents benzofuran moiety and blue color represents benzylidene moiety.

Aurones are the essential plant secondary metabolites of biologically stimulating natural products and are widely distributed in the flowers and fruits of various plants species. In addition to plant sources, aurones are also distributed in certain brown algae, bryophytes and gymnosperms class species [17,18]. Indeed, aurones occur in minute concentrations in natural sources and are therefore described as minor flavonoids that are not yet well explored. Principally, aurones act as a coloring agent, giving bright and attractive colors to flowers such as cosmos, snapdragons and some ornamental plants, etc. Therefore, aurones also play an important role in pollination, which gives them an essential role in crop production from an agricultural point of view [19]. Aurones are distributed in a limited number of genera, such as *Asteraceae*, *Anacardiaceae*, *Cactaceae*, *Cyperaceae*, *Fabaceae*, *Gesneriaceae*, *Oxalidaceae*, *Moraceae*, *Plumbaginaceae*, *Rhamnaceae*, *Rosaceae*, *Rubiaceae* and *Scrophulariaceae*, etc. [18,20]. Also, depending on their taxonomic importance in the plant kingdom, aurones may have various skeletal substitution patterns, as shown in Figure 2. Principally, aurones can be classified as 4-hydroxyaurones, 4-deoxyaurones, penylated aurones, glycosylated aurones, epimeric mixtures (aurones in bimers or trimers), etc. Interestingly, the *Asteraceae* species is rich in 4-deoxyaurones, for instance Sulfuretin, Sulfurein, Maritimetin, Maritimein, Leptosidin and Leptosin, etc. [18,21]. In addition, the flowers of the *Asteraceae* species are rich in aurone glycosides such as di-glucosides and acetylated aurone glucosides. In particular, the aurones isolated from the sunflower family or the *Bidens* genus interestingly showed hydroxylation in the 6-position (ring A) and the 3- and 4-positions (in ring B), but not in the 4-position of the aurone skeleton [20]. Moreover, the *Moraceae* species was rich in structurally distinct auronols, prenylated and geranylated aurones [22]. Interestingly, various aurone dimers such as flavanone-auronol, isoflavanone-auronol, deoxyauronol-auronol, auronol-auronol (biauronols) and other epimeric mixtures from the plant species *Anacardiaceae* and *Rhamnaceae* have also been reported [20]. Moreover, the 4-, 6-hydroxyl substitutions in the ring-A and the 4′, 3′-hydroxyl substitutions in the ring-B of aroune are most common and are related with the biosynthetic pathways. However, the skeletal substitutions of aurones depended on the biochemical reactions connected in the biosynthesis of aurones [23], which might vary by family and tropical subcontinent depending on seasonal temperatures.

## 3. Biosynthesis of Aurones

**Figure 3:**
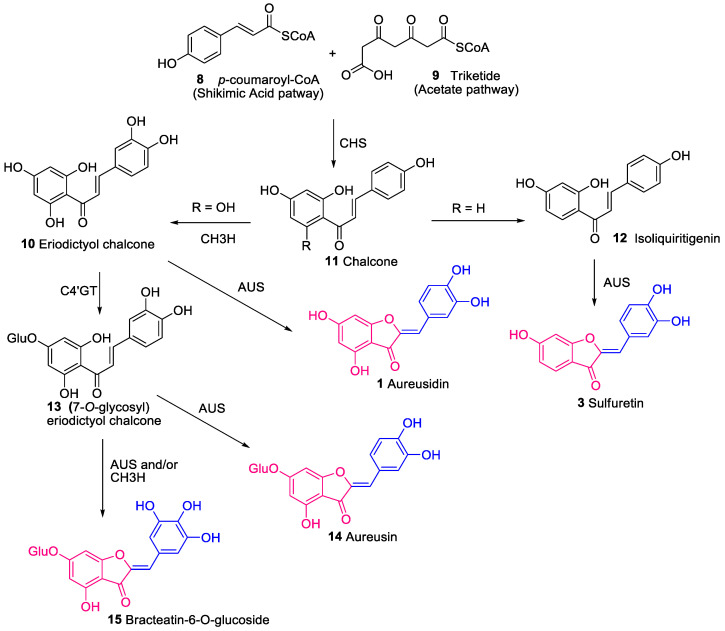
The biosynthetic approach of aurones from chalcones. Pink color represents benzofuran moiety and blue color represents benzylidene moiety.

The occurrences of secondary metabolites, for example, polyphenols, alkaloids, terpenoids, steroids, polyketides, and so on are common in the plant kingdom. In particular, secondary metabolites which are produced in plants have diverse functions such as photoprotection, enzyme modulation, defense against pathogen invasion, reproductive persistence, symbiosis and other growth-regulating defenses mechanisms. However, polyphenols represent the largest family of plant secondary metabolites formed via biogenesis pathways and are generally involved in protection against disease mechanisms and photoprotection. The biosynthesis or biogenesis of aurones in plant kingdom can be comprehensively classified into two steps: the primary step involves the synthesis of 2′-hydroxychalcones from coumaryl-CoA, and the second step involves hydroxylation and oxidative cyclization of hydroxychalcones [24,25,26]. The biogenesis of chalcones was catalyzed by chalcone synthase (CHS) via the reaction between the acetate and shikimic acid, which has been well described in several reports [27,28]. Therefore, the present section discusses the biosynthesis of aurones from chalcones in the following mechanisms as described in Figure 3. Principally, aurone biosynthesis was catalyzed by two important enzymes such as chalcone hydroxylase (CHH) and aurone synthase (AUS) [24,25]. The homolog of plant polyphenol oxidase (PPO), chalcone 3-hydroxylase (CH3H) enzyme, catalyzes the addition of hydroxyl groups to the *ortho*-position to the existing hydroxyl group on ring-B, through oxidation prototyping [21]. Likewise, the second enzyme aurone synthase (AUS) plays a crucial role in the cyclization to form benzofuran skeleton [25], while the other enzyme chalcone 4′-glucosyl transferase (C4′GT) effectively catalyzes in the formation of glycosylated aurones in the plant kingdom. Therefore, the PPO plays a key role in the oxidation and existence of diverse substitutional pattern of aurones in plant sources. 

## 4. Outline on the Concealed Pathways of Aurone Synthesis

In general, the isolation of natural products is a lengthy process and sometimes only very rare pure substances are obtained, so the structure elucidation is often insufficient. However, once the structure is confirmed, biological studies sometimes require more substance to conduct experiments and sometimes take years to establish their prominence. In such a case, medicinal chemistry is the promising avenue to synthesize and provide the desired natural compounds for clinical and therapeutic purposes without distressing the natural sources. Accordingly, the synthesis of aurones with grouped substituents worked with admittance to pharmaceutical and materials science applications. According to the literature, the highly selective synthesis of aurones can be basically divided into five synthetic routes based on the starting materials.

### 4.1. Route 1: Condensation of Benzofuran-3(2H)-one

**Scheme 1:**
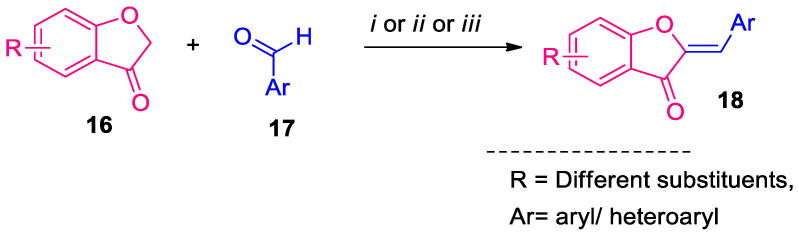
Synthesis of aurones; Reagents and conditions: (i) KOH (50 mol%), alcohol, reflux; (ii) neutral alumina, DCM (dry), N_2_, rt, overnight; (iii) Choline chloride, urea, MWI, 30 min. Pink color represents benzofuran moiety and blue color represents benzylidene moiety.

This is the simplest approach to obtain aurones from easily accessible starting materials such as benzofuran-3-one **16** and aldehydes **17** (Scheme 1). Lunven et al. [29] prepared a series of aurones **18** in 17–85% yields using a base (K_2_CO_3_)-mediated condensation of benzofuran-3-one with various aldehydes. Later, Schmitt and Handy [30] also prepared several aurones in 40–83% yields using neutral alumina-mediated Knoevenagel condensation of benzofuran-3-one **16** with various aldehydes **17**. Likewise, Taylor et al. [31] proposed a rapid synthesis of aurones using a eutectic solvent under microwave irradiation (MWI) for 30 min, resulting in 17–96% yields.

### 4.2. Route 2: Annulation of Ortho-Iodophenol

**Scheme 2:**
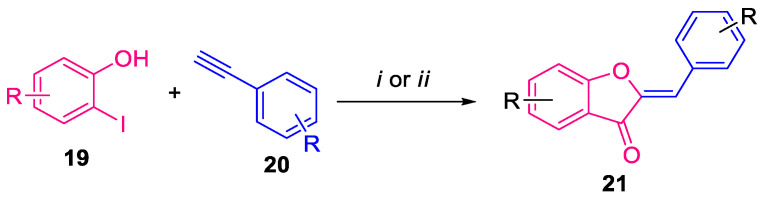
Synthesis of aurones; reagents and conditions: (i) Pd(PPh_3_)_4_ (3 mol%), Et_3_N, HCOOH, Me_2_CO, PhMe, 80 °C; (ii) Pd(OAc)_2_ (5 mol%), dppf (10 mol%), Et_3_N, CO, THF, 50 °C, 24 h. Pink color represents benzofuran moiety and blue color represents benzylidene moiety.

The second synthetic approach to aurones is the Pd-catalyzed regioselective coupling of *o*-iodophenols **19** with terminal alkynes **20** (Scheme 2) under a carbonylation source. As such, Qi and co-workers [32] reported a palladium-catalyzed synthesis of aurones **21** in 51–82% yields via an innovative carbonylation approach using formic acid as the CO source and acetic anhydride as the additive. Later, Xi et al. [33] proposed another palladium-catalyzed regioselective carbonylation reaction under Et_3_N, which provided aurones **21** in good to excellent yields (72–93%). 

### 4.3. Route 3: Cyclization of Chalcones

**Scheme 3:**
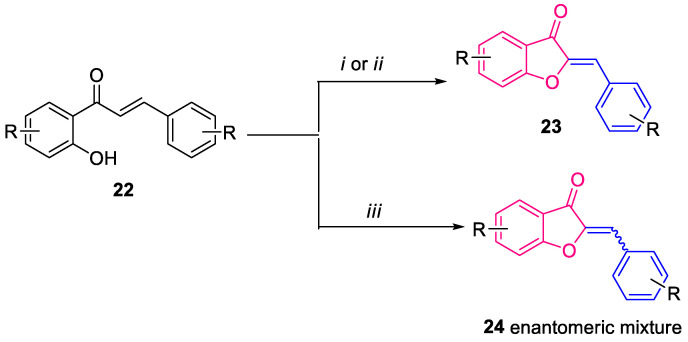
Synthesis of aurones; reagents and conditions: (i) Hg(OAc)_2_, Py, reflux, 10–15 min; (ii) CuBr_2_, DMSO, 60–90 min; (iii) Pd-Au/CeO_2_, BuOAc (2 mL), open air (1 atm), 100 °C, 24–50 h. Pink color represents benzofuran moiety and blue color represents benzylidene moiety.

This approach is similar to the biosynthesis of aurones, in which 2′-hydroxychalcones undergo dehydrogenative cyclization on exposure with oxidative agents (Scheme 3). In 2006, Agrawal and Soni [34] first proposed a convenient method for the synthesis of aurones **23** excellent yields (77–85%) via the oxidation of 2′-hydroxychalcones **22** in presence of mercury(II) acetate in pyridine under refluxed conditions for 10–15 min. Subsequently, the same research group also proposed a second oxidation method, which also succeeded in oxidizing chalcone **22** to aurones **23** in 70–80% yields by using a catalytic amount of copper(II) bromide in DMSO under refluxed conditions for 60–90 min. Later, Yatabe and co-workers [35] also developed another oxidation procedure for cyclization of chalcones to aurones **24** in 16–80% yields with <99% enantiomeric selectivity by using the heterogeneous nano-catalyst Pd-Au-supported CeO_2_. 

### 4.4. Route 4: Intramolecular Rearrangement of Oxiranes

**Scheme 4:**
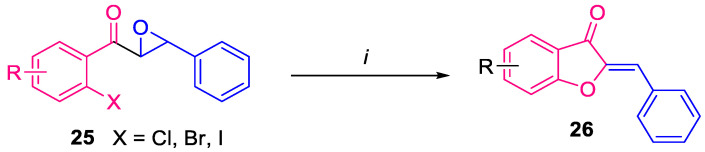
Synthesis of aurones; (i) CuI (10 mol%), 1,10-phenanthroline (20 mol%), Cs_2_CO_3_, DMF, 105 °C, N_2._ Pink color represents benzofuran moiety and blue color represents benzylidene moiety.

In this approach, oxiranes were initially prepared by the oxidation of chalcones using H_2_O_2_ (30%). Further, a copper-catalyzed tandem intramolecular ring-opening of oxiranes **25** followed by Ullman coupling [36] provided various stereoselective (*Z*)-aurones **26** in moderate to good yields (57–84%) (Scheme 4). Certainly, this is the best one-pot tandem intramolecular stereoselective approach to attain desired natural aurone analogues from inexpensive starting materials.

### 4.5. Route 5: Ring Contraction of Flavones

**Scheme 5:**
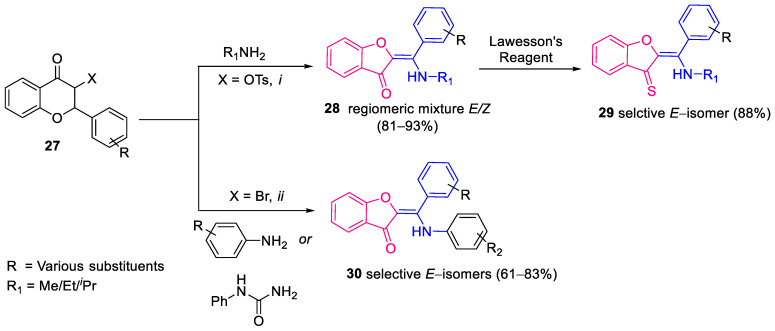
Synthesis of aurones; (i) THF, rt, 24 h; (ii) KO*^t^*Bu, CuI (5 mol%), DMF, 25 °C, 20–30 min. Pink color represents benzofuran moiety and blue color represents benzylidene moiety.

This approach enables the synthesis of hydride aurone analogues as aspects of heterocycles-assimilated aurones for the design and development of new therapeutic agents (Scheme 5). Initially, Kandioller et al. [37] proposed a ring-contraction reaction by treating 3-tosylflavones **27** with (1′-alkyl)amines to obtain the corresponding regiomeric mixture of *E*/*Z* 2′-alkylamino aurones **28** in 81–93% yields. Interestingly, upon further treatment with Lawesson’s reagent, the alkylamino-substituted aurones gave exclusively the stereoselective *E*-isomers of 3(2*H*)-thiaurones **29** in 88% yield. Likewise, Praveen and Ahmed [38] proposed a convenient approach to stereospecific *E*-aminated aurones **30** in 61–83% yields via the sequential aza-Michael addition, ring opening and subsequent ring-closing approach. This method is very facile as the 3-bromoflavones **27** provided the desired *E*-aminated aurones **30** upon treatment with amines or *N*-phenylurea in the presence of KO*^t^*Bu and CuI in DMF under mild conditions. 

## 5. Antidiabetic Potentialities of Aurones 

Aurones are the most interesting secondary metabolites of plants, since they possess diverse pharmacological activities due to structurally distinctive substitutions and possible skeletal modifications via approaches of medicinal chemistry [39]. Certainly, aurones are specific templates with compelling antioxidant potential, as the polyhydroxy substitution pattern and the conjugated benzylidene moiety play crucial roles in shielding free radicals through H-atom donor and electron-transfer mechanisms [40,41]. Hence, aurones also play a crucial role in the prevention and diagnosis of the multiple pathogenesis of various diseases such as cancers, diabetes, inflammation and neurodegenerative disorders, etc. [40]. In fact, very few studies have been reported on the prospective of aurones as antidiabetic drug developments. Consequently, the prominence of aurones and their key role in the prevention and treatment of diabetes mellitus have been summarized in this section.

**Figure 4:**
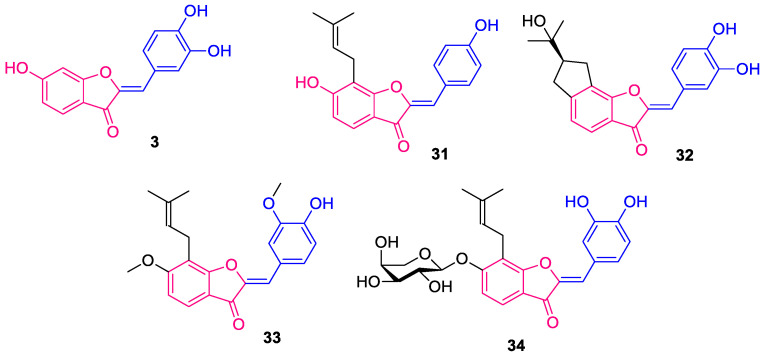
Natural aurones as antidiabetic lead agents. Pink color represents benzofuran moiety and blue color represents benzylidene moiety.

Accordingly, the intention of diabetes mellitus and the associated molecular mechanism can be classified broadly into two pathways: (i) non-enzymatic pathway and (ii) enzymatic pathway. Extensive studies are currently being conducted on the enzymatic catalysis pathways and their prevention of diabetes mellitus [42,43]. Among them, the inhibitors of α-glucosidase, aldose reductase (ALR2), diacylglycerol acyltransferase (DGAT), protein tyrosine phosphatase localized to mitochondrion 1 (PTPM1), peroxisome proliferator-activated receptor gamma (PPARγ), DRAK2 and advanced glycation end products (AGE) are excessively studied enzymatic mechanisms of diabetes [42]. Excitingly, the anural aurone, i.e., sulfuretin **3**, showed broad-spectrum antidiabetic results through various pathways (Table 1, Figure 4). As such, a study has revealed that sulfuretin **3** showed significant ALR2 activity with identical IC_50_ 1.3 µM compared to the standard drug Epalrestat [44]. Further, the study also disclosed that sulfuretin plays a crucial role in inhibiting AGE formation, with an IC_50_ 124.7 µM, which is 10-fold lower than that of the reference aminoguanidine (1231.0 µM). Another study found that sulfuretin **3** had a potential antidiabetic strategy of suppressing the molecular mechanisms of NF-κB, which is also beneficial in preventing to damage of pancreatic *β*-cells [45]. Likewise, another study also disclosed that sulferetin **3** is useful as an antidiabetic agent due to its ability to quench Maillard reactions, a non-enzymatic reaction of glucose with protein to form reversible Schiff’s base adducts [46,47].

In 2019, Zhu and co-workers [48] reported structurally interesting *C*-prenylated aurones **31** and **32** from the seed extract of *Psoralea corylifolia*. Subsequently, an in vitro enzyme inhibitory evaluation of plant metabolites was performed against diabetes targets such as diacylglycerol acyltransferase (DGAT), protein tyrosine phosphatase 1B (PTP1B) and α-glucosidase [48]. The aurone **31** showed remarkable antidiabetic activities against PTP1B (IC_50_ 11.3 µM) and DGAT (IC_50_ 35.2 µM). While the aurones **31** and **32** displayed prominent α-glucosidase enzyme inhibitory activities (Table 1, Figure 4) such as IC_50_ 73.8 and 62.1 µM, respectively.

In 2021, Chen and co-workers [49] isolated a *C*-prenylated aurone **33** from the stems of *Acanthopanax senticosus*. The subsequent in vitro α-glucosidase inhibitory study disclosed that compound **33** was beneficial as prominent antidiabetic agent with IC_50_ 64.1 µM as compared to the standard acarbose (IC_50_ 214.5 µM). A recent study [50] also revealed another interesting aurone glycoside **34** (Figure 4) from *Saussurea involucrate* and showed potent inhibitory activities against α-glucosidase enzyme with IC_50_ 47.1 µM compared to the standard acarbose.

**Figure 5:**
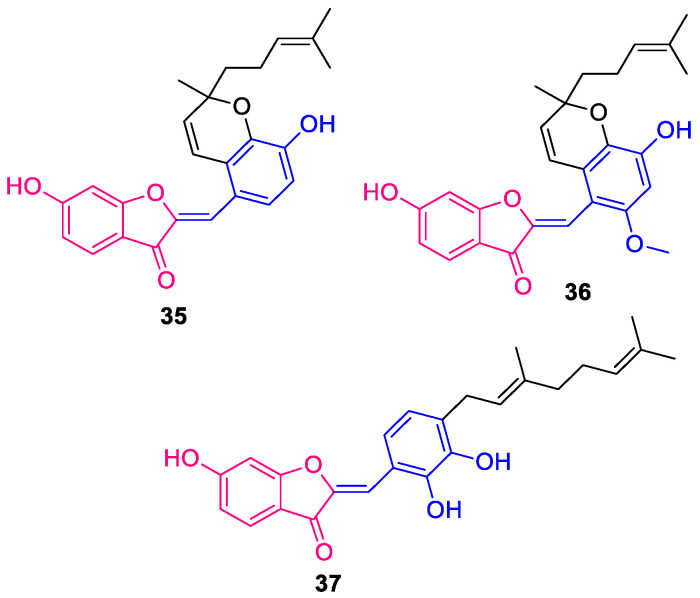
Natural geranylated aurones as antidiabetic lead agents. Pink color represents benzofuran moiety and blue color represents benzylidene moiety.

In addition, Mai and co-workers [51] isolated three structurally interesting *C*-geranylated aurones **35**–**37** (Figure 5) from the leaves of *Artocarpus altilis*. Subsequent antidiabetic bioactivity experiments revealed that the geranylated aurones **35**–**37** presented potent α-glucosidase inhibitory concentrations IC_50_ of 4.9 µM, 5.4 µM and 5.1 µM, respectively, than the standard acarbose (241.8 µM). Therefore, these natural aurones could be beneficial for the development of principal clinical antidiabetic agents; however, in vivo experimental studies and drug toxicity of aurones are still needed.

**Figure 6:**
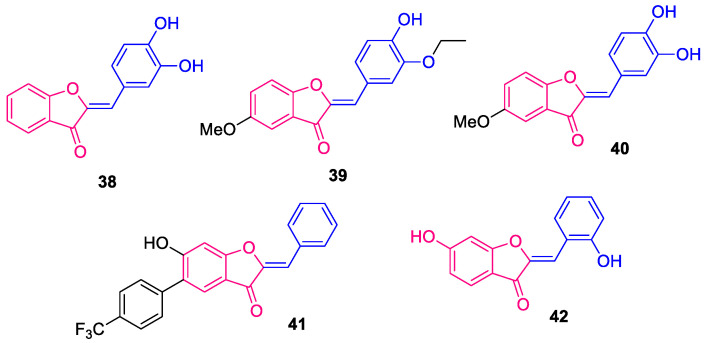
Synthesized aurones as antidiabetic lead agents. Pink color represents benzofuran moiety and blue color represents benzylidene moiety.

In the same way, Wang et al. [52] investigated a series of synthetic aurones as target of death-associated protein kinase-related apoptosis-inducing kinase-2 (DARK2) inhibitors. In a preliminary examination of the study revealed that the aurone **38** displayed significant DARK2 inhibition with an IC_50_ of 3.15 µM. Subsequently, quantified structure–activity relationship study results concealed that the aurones **39** and **40** (Figure 6) displayed superior activities with IC_50_ 0.33 µM and 0.25 µM in a dose-dependent manner and might be beneficial for antidiabetic therapeutic agents to protect islet *β*-cells from apoptosis.

Further, Sun and co-workers [53] proposed a series of 6-hydroxyaurones as target for the development of new α-glucosidase enzyme inhibitors. The results of inhibitory kinetics and molecular docking studies revealed that the aurone **41** was a potent α-glucosidase inhibitor with an IC_50_ 30.94 µM than standard acarbose (IC_50_ 50.30 µM). Interestingly, the compound **41** exhibited an identical glucose consumption-promoting activity in HepG2 cells at 1 µM as like metformin.

Also, mitoNEET is a 2Fe-2S cluster membrane protein and a key regulator of mitochondrial functions in various metabolic diseases such as cancers and obesity, etc. [54]. Also, the potent antidiabetic drugs such as rosiglitazone and pioglitazone were found to be effective mitoNEET binders, and hence, the protein mitoNEET was considered as diabetic target. Accordingly, a rationalized identification study of the mitoNEET inhibitor of mitochondrial protein revealed that aurone **42** (Figure 6) exhibited potent binding affinity K_i_ 6nM with mitoNEET [55].

**Figure 7:**
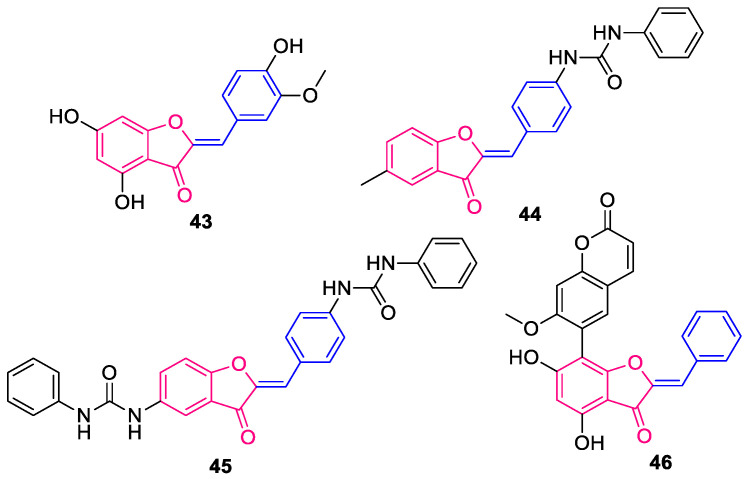
Synthesized aurones as antidiabetic lead agents. Pink color represents benzofuran moiety and blue color represents benzylidene moiety.

Later, Roshanzamir and co-authors [56] proposed a structure-optimized study of a series of aurones to evaluate their in vitro and in silico biological activities against porcine pancreatic α-amylase (PPA). Accordingly, the study revealed that the hydroxyl groups on both phenyl rings of the aurone are crucial for the formation of hydrogen bonding interactions with the catalytic residues of the binding target and for their increased inhibitory activities. Also, the aurone (**43**) with 4,6-dihyroxybenzofuranone and a 4′-hydroxyl group on the benzylidene (Figure 7) showed important binding interactions with amino acid residues in the active sites of the target PPA. Therefore, the aurone **43** showed an interesting in vitro enzyme inhibitory IC_50_ 40.25 µM of PPA activity (Table 1) and could be beneficial as a leading drug template for future developments of anti-diabetic drugs. In addition, a recent study [57] also reflected a series of synthesized phenylureidoaurones as targets for the development of effective anti-diabetic therapeutic agents. Consequently, the conducted enzyme inhibitory and computational study identified two phenylureidoaurones **44** and **45** (Figure 7) with strategic anti-diabetic results. The aurone **44** demonstrated principal inhibitory activity on α-amylase with an IC_50_ 142.0 µM, and a moderate α-glucosidase inhibition IC_50_ 292.7µM compared to standard acarbose. However, the bis-phenylureido aurone **45** showed the highest α-glucosidase inhibition with an IC_50_ of 6.6 µM and could be beneficial for the development of lead anti-diabetic drugs.

**Table 1:** Summary of aurones and analogue aurones listed as antidiabetic lead agents.

Compound	Antidiabetic Target	IC_50_	Ref.
**Natural aurones**			
Sulfuretin (**3**)	ALR2	1.3 µM	[44]
	AGE	124.7 µM	[44]
	NF-κB	-	[45]
	Millard reaction (non-enzyme) inhibitor	-	[46]
(*Z*)-6-Hydroxy-2-(4-hydroxybenzylidene)-7-(3-methylbut-2-en-1-yl)benzofuran-3(2*H*)-one (**31**)	PTP1B	11.3 µM	[48]
	DGAT	35.2 µM	
	α-glucosidase	73.8 µM	
(*R*,*Z*)-2-(3,4-Dihydroxybenzylidene)-7-(2-hydroxypropan-2-yl)-7,8-dihydro-2*H*-indeno[4,5-*b*]furan-3(6*H*)-one (**32**)	α-glucosidase	62.1 µM	[48]
(2*Z*)-2-[(4′-Hydroxy-3′-methoxyphenyl) methylene]-6-methoxy-7-prenyl-3(2*H*)-benzofurane (**33**)	α-glucosidase	64.1 µM	[49]
Licoagroaurone-6-O-α-L-arabinopyranoside (**34**)	α-glucosidase	47.1 µM	[50]
Altilisin H (**35**)	α-glucosidase	4.9 µM	[51]
Altilisin I (**36**)	α-glucosidase	5.4 µM	[51]
Altilisin J (**37**)	α-glucosidase	5.1 µM	[51]
**Synthetic aurones**			
(*Z*)-2-(3,4-Dihydroxybenzylidene)benzofuran-3(2*H*)-one (**38**)	DARK2	3.15 µM	[52]
(*Z*)-2-(3-Ethoxy-4-hydroxybenzylidene)-5-methoxybenzofuran-3(2*H*)-one (**39**)	DARK2	0.33 µM	[52]
(*Z*)-2-(3,4-Dihydroxybenzylidene)-5-methoxybenzofuran-3(2*H*)-one (**40**)	DARK2	0.25 µM	[52]
(*Z*)-2-Benzylidene-5-(4-fluorophenyl)-6-hydroxybenzofuran-3(2*H*)-one (**41**)	α-glucosidase	30.94 nM	[53]
(*Z*)-6-Hydroxy-2-(2-hydroxybenzylidene)benzofuran-3(2*H*)-one (**42**)	mitoNEET	0.62 nM	[55]
(*Z*)-4,6-dihydroxy-2-(4-hydroxy-3-methoxybenzylidene)benzofuran-3(2*H*)-one (**43**)	PPA	40.25 µM	[56]
(*Z*)-1-(4-((5-methyl-3-oxobenzofuran-2(3*H*)-ylidene)methyl)phenyl)-3-phenylurea (**44**)	α-glucosidase	292.7 µM	[57]
(*Z*)-1-(4-((5-(3-Phenylureido)-3-oxobenzofuran-2(3*H*)-ylidene)methyl)phenyl)-3-phenylurea (**45**)	α-amylase	142.0 µM	[57]
	α-glucosidase	6.6 µM	[57]
(*Z*)-6-(2-benzylidene-4,6-dihydroxy-3-oxo-2,3-dihydrobenzofuran-7-yl)-7-methoxy-2*H*-chromen-2-one (**46**)	α-glucosidase	3.55 µM	[58]
	α-amylase	10.97 µM	[58]
**Analogue aurones**			
(*Z*)-4-(5-((3-oxobenzo[*b*]thiophen-2(3*H*)-ylidene)methyl)furan-2-yl)benzoic acid (**47**)	PTPM1	11.8 µM	[59]
(*E*)-5,6-dimethoxy-2-(2-(2-(thiophen-2-yl)ethoxy) benzylidene)-2,3-dihydro-1*H*-inden-1-one (**48**)	PPAR-γ	0.61 µM	[60]
(*E*)-2-(4-(2-(5-ethylpyridin-2-yl)ethoxy)benzylidene)-5,6-dimethoxy-2,3-dihydro-1H-inden-1-one (**49**)	PPAR-γ	1.20 µM	[60]

In addition, Sun and co-workers [58] also considered four series of natural coumarin, i.e., umbelliferon integrated synthesized hybrids, as targets for the development of novel antidiabetic agents. As a result of the in vitro enzyme inhibitory studies and its kinetic analysis, the coumarin–aurone hybrid **46** was found to have strategic α-amylase and α-glucosidase inhibition activities with IC_50_ 10.97 and 3.55 µM, respectively. Moreover, the coumarin–aurone hybrid **46** presented both α-glucosidase and α-amylase equally as the standard acarbose drug and also exhibited HepG2 cell-based glucose consumption-promoting activity in insulin and non-insulin resistant models. Therefore, this dual-pharmacophore-based scaffold **46** could be beneficial for drug developments to tackle the complex pattern disorders such as diabetes mellitus.

**Figure 8:**
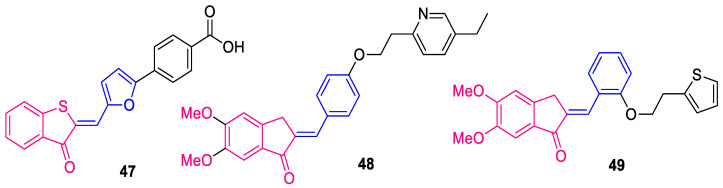
Analogue aurones as anti-diabetic lead agents. Pink color represents benzofuran moiety and blue color represents benzylidene moiety.

Owing to the significant antidiabetic activities of natural aurones, some examples of aurone analogues have also been identified as targets of inhibitory agents via in silico and synthetic approaches. Accordingly, Park et al. [59] reported a quantified in silico study to identify protein tyrosine phosphatase mitochondrial 1 (PTPM1) inhibitors for the treatment of type II diabetes. The computational screening of inhibitors of human PTPM1 conceals that an analogue aroune **47** (Figure 8) exhibited potent IC_50_ 11.8 µM concentrations, and further clinical experimental studies are needed to establish its therapeutic potency. In addition, Chaturvedi and co-workers [60] reported various series of synthetic analogues as a target for the development of effective antidiabetic lead agents. Accordingly, the in vitro inhibitory and molecular docking studies showed that the analogue of aurones **48** and **49** exhibited strong IC_50_ 0.62 µM and 1.20 µM concentrations, respectively, against peroxisome proliferator-activated receptor-γ (PPAR-γ).

### Glycosidase Activity of Aurones

Glycosidase enzymes are important for the catalytic mechanisms of hydrolysis of glycosidic bonds of polysaccharides. To date, more than 50 families of glucosidases have been compiled in the literature based on amino-acid sequences [61,62]. However, based on the catalytic activity, glycosidases can be categorized into (i) endo-glycosidase that hydrolyze the internal glycosidic bonds of oligosaccharides and the other (ii) exo-glycosidases that hydrolyze a single monosaccharide (at control rates) from the non-reducing terminus of the oligosaccharide [61]. Glycosidases are particularly degradative enzymes for the digestion of extracellular carbohydrates into monosaccharides. Moreover, glycosidase also accomplish another important degradative intracellular function, namely the catabolism of polysaccharides, as a physiological function of the energy source [62]. Therefore, glycosides are responsible for both extracellular and intracellular activities and are necessary for carbohydrate and glycoprotein degradation.

**Figure 9:**
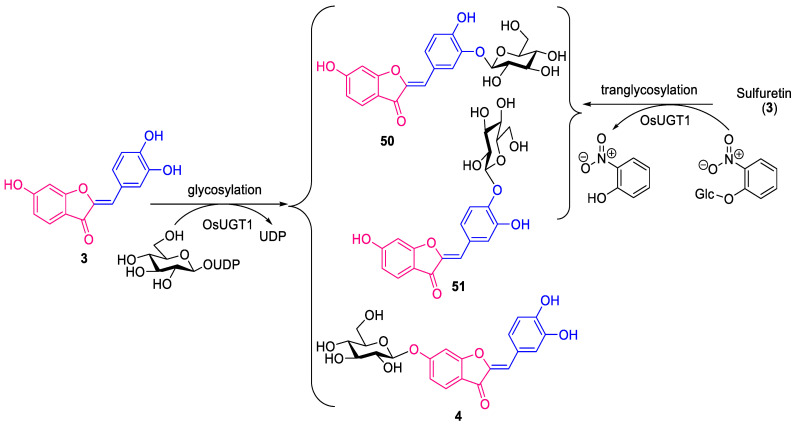
The biocatalytic (OsUGT1) glycosylation and transglycosylation pattern of aurones. Pink color represents benzofuran moiety and blue color represents benzylidene moiety.

Interestingly, a recent study [63] revealed that aurone showed significant glycosidase activity in their in vitro enzymetic model experiments. Accordingly, an analogue aurone uridine diphosphate glycosyltransferase (OsUGT1) was isolated from the medicinal plant, *Ornithogalum saundersiae*, and prepared as a biocatalyst for the glycosylation reaction. Later, a representative aurone sulfuretin was subjected to glycosylation with the sugar donor UDP-O-glucose in the presence of catalyst OsUGT1, as characterized in Figure 9. Subsequently, through purification and characterization, it was found that the glycosylation reaction yields three regioselective monoglycosides such as sulfuretin 3′-*O*-glucoside (**50**), sulfuretin 4′-*O*-glucoside (**51**) and sulfuretin 6-*O*-glucoside (**4**). Further, the catalytic glycosidase ability of OsUGT1 was reexamined through a transglycosylation approach using an alternative ortho-nitrophenyl-*β*-*O*-glucoside (*o*NP-*β*-Glc). Accordingly, intermolecular transglycosylation between sulfuretin and *o*NP-*β*-Glc afforded the corresponding monoglycosides (**50** and **51**) and deglucosylated *O*-nitrophenol (*o*NP). Overall, the study reveals the biocatalytic application of OsUGT1 and the biosynthesis of aurone glucosides.

Indeed, glycosidase activity and anti-diabetic activity are two different parameters, but aurone scaffolds showed interesting results in both studies. The authors of the current study hypothesize that the aurone may be beneficial in delaying glucose digestion since; as discussed above, aurones readily bind glucose and forms aurone glycosides via enzymatic biosynthetic pathways, while in the currently practiced anti-diabetic diagnosis tactics, delayed glucose digestion is one of the key processes of blood glucose tolerance. Therefore, concurrent antidiabetic studies of aurones are required for their capabilities in delayed sugar digestion experiments.

## 6. Conclusions and Future Perspectives

In summary, aurone is an interesting and skillful template for diverse biological activities and potential materials science applications. Additionally, various studies have shown that aurones play a strategic physiological role in the inhibition and prevention of tumors and certain types of cancers. Equally, plant aurones act as proficient anti-oxidants as they are rich in poly-hydroxylated and active-benzylidene moieties which play a crucial role in shielding exogenous and endogenous free-radicals. Therefore, the persuasive antioxidant properties of aurones could be helpful in retarding the metabolic pathways of diabetes mellitus. In addition, the studies summarized above on the anti-diabetic potentialities of aurones and their analogues also showed promising results on impaired glucose amelioration and inhibition of the various diabetic molecular signaling pathways. In particular, sulfuretin **3** showed promising antidiabetic activities through targeting various chronic metabolic signaling pathways. In addition, the *C*-gernalylated aurones, i.e., Altilisin H-I (**35**–**37**), showed significant α-glucosidase inhibition IC_50_ 4.9–5.4 µM and could be valuable for the development of antidiabetic drug leads. Accordingly, aurones are considered as key scaffolds in delaying glucose digestion and absorption and are the most important antidiabetic approach in postprandial hyperglycemia. Sequentially, the aurones with potential α-glucosidase inhibitors are also constructively effective in inhibiting or preventing various metabolic diseases such as cancers, viral diseases, etc. However, today there is only very limited clinical evidence on antidiabetic studies of aurones, which are also exclusively based on cell-based studies. Further imminent studies are required to develop effective anti-diabetic aurones and to address this complex-patterned metabolic disorder. Considering this, the current review could be useful as a template for future design and development of biologically important aurones.

## Data Availability

No new data were created or analyzed in this study. Data sharing is not applicable to this article.
